# Tumor-associated lymphatic vessel density is a postoperative prognostic biomarker of hepatobiliary cancers: a systematic review and meta-analysis

**DOI:** 10.3389/fimmu.2024.1519999

**Published:** 2025-01-07

**Authors:** Jin Li, Yu-Bo Liang, Qing-Bo Wang, Yu-Kai Li, Xing-Ming Chen, Wan-Ling Luo, Yawhan Lakang, Zi-Sheng Yang, Yan Wang, Zhi-Wei Li, Yang Ke

**Affiliations:** ^1^ Department of Hepatobiliary Surgery, The Second Affiliated Hospital, Kunming Medical University, Kunming, China; ^2^ Department of Pathology, The Second Affiliated Hospital, Kunming Medical University, Kunming, China; ^3^ Division of Hepatobiliary and Pancreatic Surgery, Department of Surgery, The First Affiliated Hospital, Zhejiang University School of Medicine, Hangzhou, China; ^4^ Department of Surgical Education and Research, The Second Affiliated Hospital, Kunming Medical University, Kunming, China; ^5^ Yunnan Yunke Bio-Technology Institution, Kunming, China

**Keywords:** bile duct cancer, hepatocellular carcinoma, liver cancer, lymphangiogenesis, pancreatic cancer, prognosis, surgery

## Abstract

**Purpose:**

This study aimed to investigate whether tumor-associated lymphatic vessel density (LVD) could predict the survival of patients with hepato-biliary-pancreatic (HBP) cancers after radical resection.

**Methods:**

A systematic search was conducted using PubMed, Embase, and Cochrane Library from the inception to July 31, 2024 for literature that reported the role of LVD in overall survival (OS) and recurrence-free survival (RFS) of patients with HBP cancers after radical resection.

**Results:**

Ten studies with 761 patients were included for the meta-analysis. The results indicated that a higher level of LVD was associated with worse OS (hazard ratio, HR = 2.87, 95% CI 1.63 to 5.04) and worse RFS (HR = 3.18, 95% CI 1.41 to 7.17) in HBP cancers. Subgroup analysis based on pathological types revealed that a higher level of LVD was significantly related to worse OS in hepatocellular carcinoma (HCC) (HR = 2.35, 95% CI 1.16 to 4.78), cholangiocarcinoma (HR = 4.65, 95% CI 1.70 to 12.70), and gallbladder cancer patients (HR = 4.64, 95% CI 1.37 to 15.71). The levels of LVD were not significantly associated with OS in pancreatic adenocarcinoma patients after radical resection (HR = 1.08, 95% CI 0.61 to 1.89). Similarly, a higher level of LVD was significantly associated with worse RFS in HCC (HR = 1.92, 95% CI 1.01 to 3.65) and cholangiocarcinoma patients (HR = 4.54, 95% CI 2.10 to 9.83).

**Conclusions:**

A higher level of LVD was a biomarker for the prediction of worse OS and RFS in patients with hepatobiliary cancers after radical resection.

**Systematic review registration:**

https://www.crd.york.ac.uk/prospero/, identifier CRD42024571167.

## Introduction

Hepato-biliary-pancreatic (HBP) cancers are a group of malignancies in digestive organs, including hepatocellular carcinoma (HCC), cholangiocarcinoma, gallbladder cancer, and pancreatic cancer (1). The HBP cancers are the second frequent digestive cancers (7.5% of total new cancer cases following colorectal cancer 9.6%) and rank the first in the leading causes of digestive cancer-related death (13.4% of total cancer deaths) in 2022 (2, 3). Although radical resection is the optimal treatment for early HBP cancers, the outcomes of this treatment remain unsatisfactory (4). Identification of novel biomarkers for the prognosis of HBP cancers is crucial and urgent in improving the surgical outcomes of patients with HBP cancers (5).

The HBP system produces the largest amount of lymph in our body, accounting for more than 50% of lymph passing through the thoracic duct (6, 7). The lymphatic vasculature in the HBP system functions to maintain fluid homeostasis, immune surveillance through transportation of various immune cells and antigens to lymph nodes, and lipid metabolism through transportation of lipids to the systemic circulation (6, 7).

Tumor-associated lymphangiogenesis is the process of new lymphatic vessel formation in tumor and recently it is a hot topic in the cancer research field (8–11). During the development of HBP cancers, tumor-associated lymphatic vessels emerge and connect the tumor with the enormous lymphatic vasculature in the HBP system (12). The extent of tumor-associated lymphangiogenesis can be quantified by lymphatic vessel density (LVD) through microscopy (13). Some studies compared the role of tumor-associated LVD in the prognosis of patients with HBP cancers after radical resection. Several studies have reported that a higher level of LVD is significantly associated with worse survival of patients with HBP cancers after radical resection (14–20), while other studies have shown that the levels of LVD are not associated with the prognosis of patients with HBP cancers after radical resection (21–23). These previous studies, however, were performed in a single center (14–17, 20–23) or two centers (18, 19), and with a relatively small sample size (the largest sample size is 114) (14–23). Therefore, although the apparent relevance of lymphangiogenesis in healthy and disease conditions of the HBP system, whether tumor-associated LVD could be valuable for the prognosis of HBP cancers after radical resection remains inconclusive.

In this study, we performed a systematic review and meta-analysis to clarify whether the levels of tumor-associated LVD could predict the survival of patients with HBP cancers after radical resection.

## Methods

The systematic review and meta-analysis were conducted following the Preferred Reporting Items for Systematic Reviews and Meta-Analyses (PRISMA) statement (24). The protocol of the study was registered in an official repository (PROSPERO CRD42024571167).

### Search strategy

PubMed, Embase, and Cochrane Library were systematically searched for all relevant literature on the association between LVD and survival outcomes in patients with HBP cancers after radical resection from the inception of databases to July 31, 2024. The search terms for HCC, cholangiocarcinoma, gallbladder cancer, and pancreatic cancer are shown in **Supplementary Table S1**. There was no filter for geographical location, study types, or language. Additionally, manual search of reference list of the retrieved literature was conducted.

### Article selection

All relevant publications were screened and examined by four investigators (J.L., Y.B.L., Q.B.W., and Y.K.L.) independently and eligible studies were identified by reviewing the titles, abstracts, and full texts of the articles. The inclusion criteria were (a) studies focusing on HCC, cholangiocarcinoma, gallbladder cancer, or pancreatic cancer patients with radical resection; (b) with immunohistochemistry data of LVD measurement in tumors; (c) dividing patients into two groups with high and low levels of LVD in the tumors, based on a specified cut-off value; (d) reporting the association between the levels of LVD and overall survival (OS) and/or recurrence-free survival (RFS) of patients. The exclusion criteria included (a) duplication, only the study with the largest sample size was included if the same population was used in multiple studies; (b) non-clinical studies: e.g., case report, conference abstract, review, meta-analysis, or cellular and/or animal experiment; (c) an article without full text available.

### Data extraction

For each included article, the relevant information was extracted by two independent reviewers (J.L. and Y.B.L.) in predefined formats, including the first author’s name, publication year, country/region, pathological diagnosis, number of patients, cut-off value of LVD, sex, mean/median age, differentiation grade, T status, N status, TNM stage, version of TNM stage, median follow-up, OS, RFS, hazard ratio (HR), and 95% confidence interval (CI). If HR and 95% CI were not directly available, they were calculated from Kaplan-Meier curves using Tierney’s method (25). Unavailable raw data were tried to obtain from the corresponding authors of articles.

### Quality assessment

Quality assessment was conducted by two independent reviewers (J.L. and Y.B.L.) based on the Newcastle-Ottawa Quality Assessment Scale (NOS) (26). Studies with scores of 7-9, 4-6, or ≤ 3 were considered to be a good, fair, or low quality (26). During the processes of article selection, data extraction, and quality assessment, any discrepancies between independent reviewers were resolved by discussion with a senior reviewer (Y.K., Z.W.L., or Y.W.).

### Statistical analysis

The potential association of a higher level of LVD with the OS and RFS of patients with HBP cancers after radical resection was evaluated by HR and 95% CI, which were compared with those with a lower level of LVD. A pooled HR greater than 1 and a 95% CI that did not cross 1 indicated that patients with a higher level of LVD in tumor had a worse survival outcome after radical resection compared to those with a lower level of LVD in HBP cancers. All patients were stratified, based on the pathological types and their OS and PFS were analyzed. The data about the survival of patients in eight studies selected were analyzed by the Cox proportional hazards regression (14–19, 21, 23), and those in other two studies selected were analyzed by the Kaplan-Meier curve and log-rank test (20, 22).

The association between LVD and clinicopathological characteristics was evaluated, based on quantitative data of the pooled standardized mean difference (SMD) and 95% CI of LVD synthesized from all studies using Cohen’s d method (27). A pooled SMD greater than 0 and its 95% CI that did not cross 0 indicated that a higher level of LVD was associated with higher T stages, positive lymph node metastasis, or poor differentiation stages.

The association between the levels of LVD and clinicopathological characteristics was also analyzed for categorized data of the aggregated odds ratio (OR) and 95% CI. A pooled OR greater than 1 and its 95% CI that did not cross 1 indicated that a higher level of LVD was associated with higher T stages, positive lymph node metastasis, or poor differentiation stages.

The I-square (I^2^) test was utilized to assess the heterogeneity of the meta-analysis results, with a threshold of 50% considered significant heterogeneity (8). In cases where I^2^ exceeded 50%, the random effects model was chosen to calculate the combined estimates, otherwise, the fixed effects model was applied (8). Potential publication bias was estimated using funnel plots and further evaluated by Begg’s test and Egger’s test (8). A *P*-value of less than 0.05 was regarded as statistically significant. All the statistical analyses were performed using the Stata 18.0 software (Stata, Texas, USA).

## Results

### Study selection

**Figure 1:**
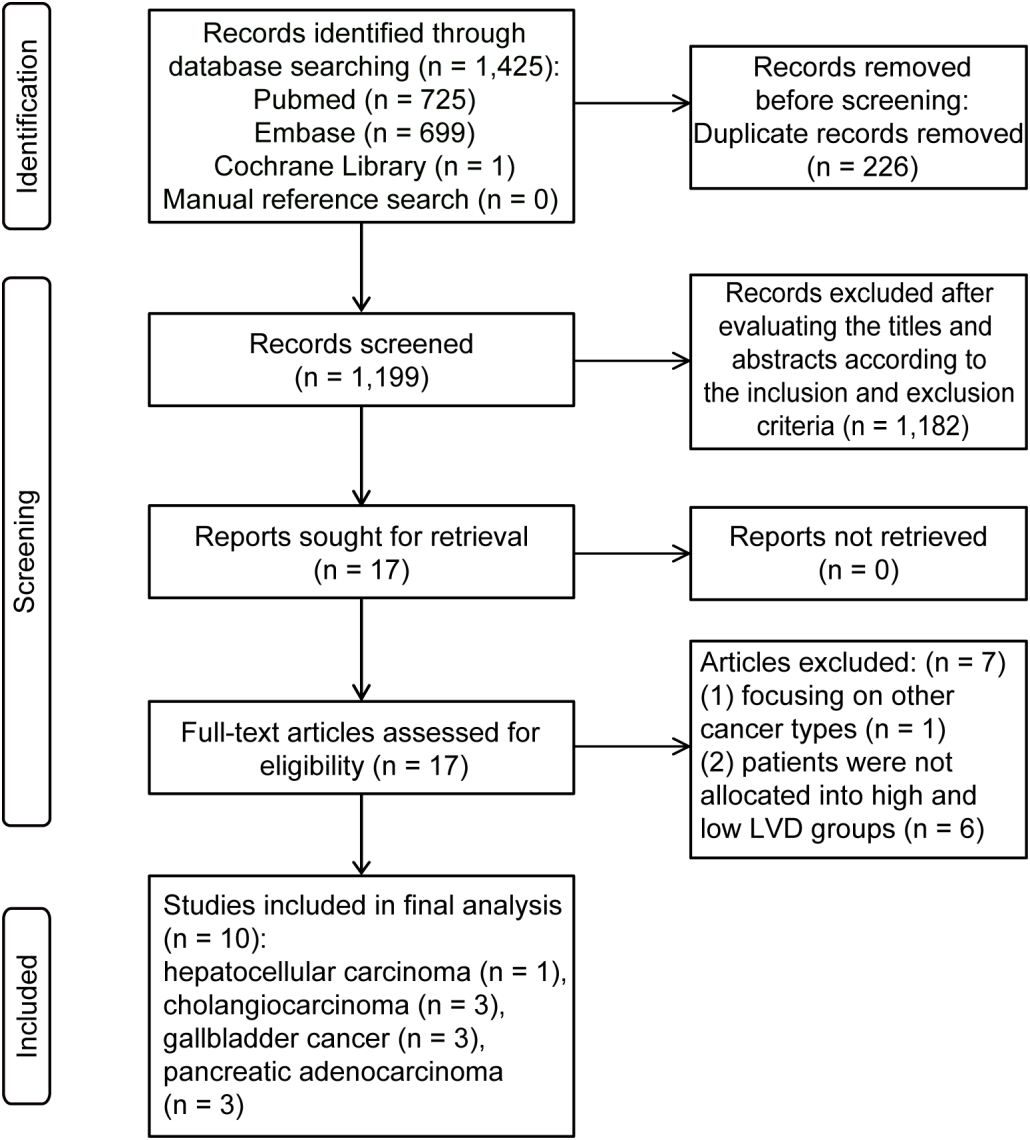
A flow chart of the process of study search and selection process.

The process of literature search, screening and identification is shown in **Figure 1**. A total of 1,425 articles were obtained from PubMed (725), Embase (699), and Cochrane Library (1). After the removal of 226 duplications, 1,199 records remained. Subsequently, 1,182 studies with irrelevant subjects were excluded after the title and abstract screening. The remaining 17 studies and their full text were evaluated according to the inclusion and exclusion criteria. An additional 7 studies were excluded for focusing on other cancer types (n = 1), or because they involved patients without high and low levels of LVD (n = 6). Ultimately, 10 retrospective studies were included in the meta-analysis with one for HCC (14), three for cholangiocarcinoma (15–17), three for gallbladder cancer (18–20), and three for pancreatic cancer (21–23). Eight studies had high quality (14–18, 20–22), and two studies had fair quality (19, 23) (**Supplementary Table S2**).

### Study and patient characteristics

The baseline characteristics of the 10 included studies are shown in **Table 1**. The studies covered 14 years (2005-2019). Four studies were conducted in Germany (14, 16, 17, 22), four in China (15, 18–20), one in Japan (21), and another one in Brazil (23). A total of 761 patients were included, with 375 male (49.3%) and 386 female (50.7%), and a median/mean age ranged from 52.6 to 67.0 years. The numbers of patients with HCC, cholangiocarcinoma, gallbladder cancer and pancreatic cancer was 60, 280, 190, and 231, respectively. The levels of LVD in these 10 studies were evaluated by immunohistochemistry after staining with anti-D2-40 (a.k.a. podoplanin) on lymphatic endothelial cells. Patients were divided into two groups with high and low levels of LVD, based on median LVD value in one study (19), mean LVD value in seven studies (14–18, 20, 23), and a specific cut-off value in the remaining two studies (21, 22). Among the 10 studies, five studies reported the number of patients with high and low levels of LVD in tumor, including 187 patients (45.9%) with high levels of LVD and 220 patients (54.1%) with low levels of LVD (14–18), while other five did not report the number of patients but reported HR and 95% CI about the role of LVD in survival (19–23). The median follow-up periods ranged from 11.5 to 28.0 months.

**Table 1:** The characteristics of patients with hepato-biliary-pancreatic cancers with higher and lower levels of lymphatic vessel density after radical resection.

Ref.	Publication year	Country/region	Pathological diagnosis	No. of patient (high vs. low)	Cut-off value	No. of Male (%)	Mean/median age (yr)	Differentiation grade (well/moderate/poor)	T status (T1/T2/T3/T4)	N status (negative/positive)	TNM (I/II/III/IV)	TNM version	Median follow-up (m)
Thelen A et al. (14)	2009	Germany	Hepatocellular carcinoma	24 vs. 36	22.9/HPF	42 (70.0)	67.0	9/39/12	3/14/30/13	58/2	NA	6	28.0
Thelen A et al. (16)	2008	Germany	Hilar cholangiocarcinoma	34 vs. 26	10.0/HPF	30 (50.0)	61.5	7/39/14	0/21/39/0	36/24	NA	6	28.0
Thelen A et al. (17)	2010	Germany	Intrahepatic cholangiocarcinoma	46 vs. 68	2.97/HPF	49 (43.0)	61.5	2/82/30	16/26/55/17	61/53	11/17/82/4	6	11.5
Sha M et al. (15)	2019	China	Intrahepatic cholangiocarcinoma	50 vs. 56	12.0/HPF	62 (58.5)	60.0	Well or moderate/poor: 52/54	NA	58/48	33/15/10/48	NA	22.1
Chen Y et al. (19)	2011	China	Gallbladder cancer	72*	13.67/HPF	27 (37.5)	62.7	NA	NA	NA	NA	NA	18.3
Wang W et al. (18)	2012	China	Gallbladder adenocarcinoma	33 vs. 34	11.4/HPF	19 (28.4)	52.6	27/21/19	NA	36/31	NA	7	NA
Jiang L et al. (20)	2018	China	Gallbladder adenocarcinoma/other gallbladder cancer^¤^	51*	7.0/HPF	20 (39.2)	59.0	20/15/16	Tis-T2/T3-T4: 15/36	24/27	0-II/III-IV: 12/39	7	NA
Sipos B et al. (22)	2005	Germany	Pancreatic adenocarcinoma^£^	98*	NA	44 (44.9)	63.0	14/55/29	1/4/74/19	28/70	3/21/39/35	6	NA
Kurahara H et al. (21)	2010	Japan	Pancreatic adenocarcinoma^¢^	70*	NA	48 (68.6)	66.7	27/37/6	2/4/62/2	16/54	I+II/III+IV: 58/12	6	NA
Zorgetto VA et al. (23)	2013	Brazil	Pancreatic adenocarcinoma^¥^	63*	15.0/HPF	34 (54.0)	62.0	18/33/12	NA	23/40	NA	NA	NA

HPF, high-power field in microscopy; m, months; NA, not available; TNM, tumor node metastasis classification; ¤, the study included 23 patients with gallbladder adenocarcinoma and 28 patients with other pathological types of gallbladder cancers; £, the pancreatic adenocarcinomas located either in the head or in the body/tail of the pancreas, however the specific number was not provided; ¢, the pancreatic adenocarcinomas located only in the head of the pancreas; ¥, the specific locations of the pancreatic adenocarcinoma were not provided; *, the total number of patients with high and low levels of lymphatic vessel density.

### Overall survival

**Figure 2:**
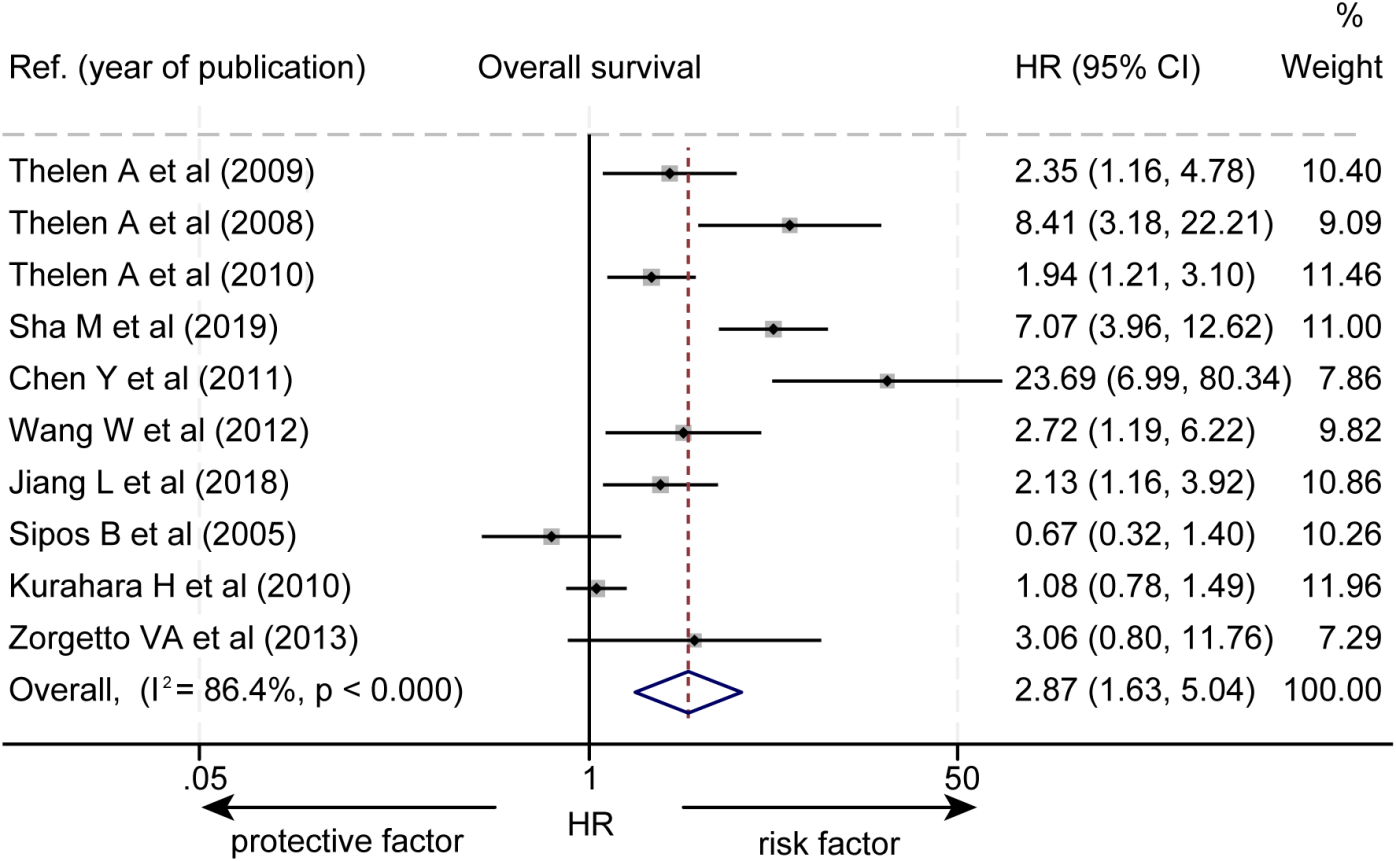
Forest plot of the impact of levels of LVD on overall survival of patients with hepato-biliary-pancreatic cancers after radical resection. CI, confidence interval; HR, hazard ratio; LVD, lymphatic vessel density.

The effect of LVD on OS was assessed in 10 studies (**Table 2**) (14–23). Seven studies reported that a higher level of LVD in HBP cancers was significantly associated with worse OS (14–20). In contrast, three studies reported that levels of LVD in tumor were not significantly associated with OS (21–23). Data pooled from these 10 studies exhibited that a higher level of LVD in tumor was significantly associated with worse OS (HR = 2.87, 95% CI 1.63 to 5.04, **Figure 2**).

**Table 2:** The overall survival of patients with hepato-biliary-pancreatic cancers with higher and lower levels of lymphatic vessel density after radical resection.

Ref.	Publication year	Group	Overall survival		
			One-year	Three-year	Five-year
Thelen A et al. (14)	2009	High	62.00%	24.00%	24.00%
		Low	74.00%	63.00%	56.00%
Thelen A et al. (16)	2008	High	62.70%	24.40%	7.00%
		Low	95.20%	90.50%	76.40%
Thelen A et al. (17)	2010	High	49.10%	9.70%	6.50%
		Low	68.20%	38.30%	31.00%
Sha M et al. (15)	2019	High	20.00%	0.00%	0.00%
		Low	76.80%	66.80%	48.00%
Chen Y et al. (19)	2011	High	100.00%	70.00%	16.00%
		Low	18.00%	5.00%	5.00%
Wang W et al. (18)	2012	High	20.00%	NA	NA
		Low	40.00%	NA	NA
Jiang L et al. (20)	2018	High	47.56%	22.70%	22.70%
		Low	67.00%	59.77%	59.77%
Sipos B et al. (22)	2005	High	64.15%	20.56%	20.56%
		Low	48.20%	12.24%	12.24%
Kurahara H et al. (21)	2010	High	NA	NA	3.50%
		Low	NA	NA	33.20%
Zorgetto VA et al. (23)	2013	High	42.96%	42.96%	21.74%
		Low	20.62%	5.00%	5.00%

**Figure 3:**
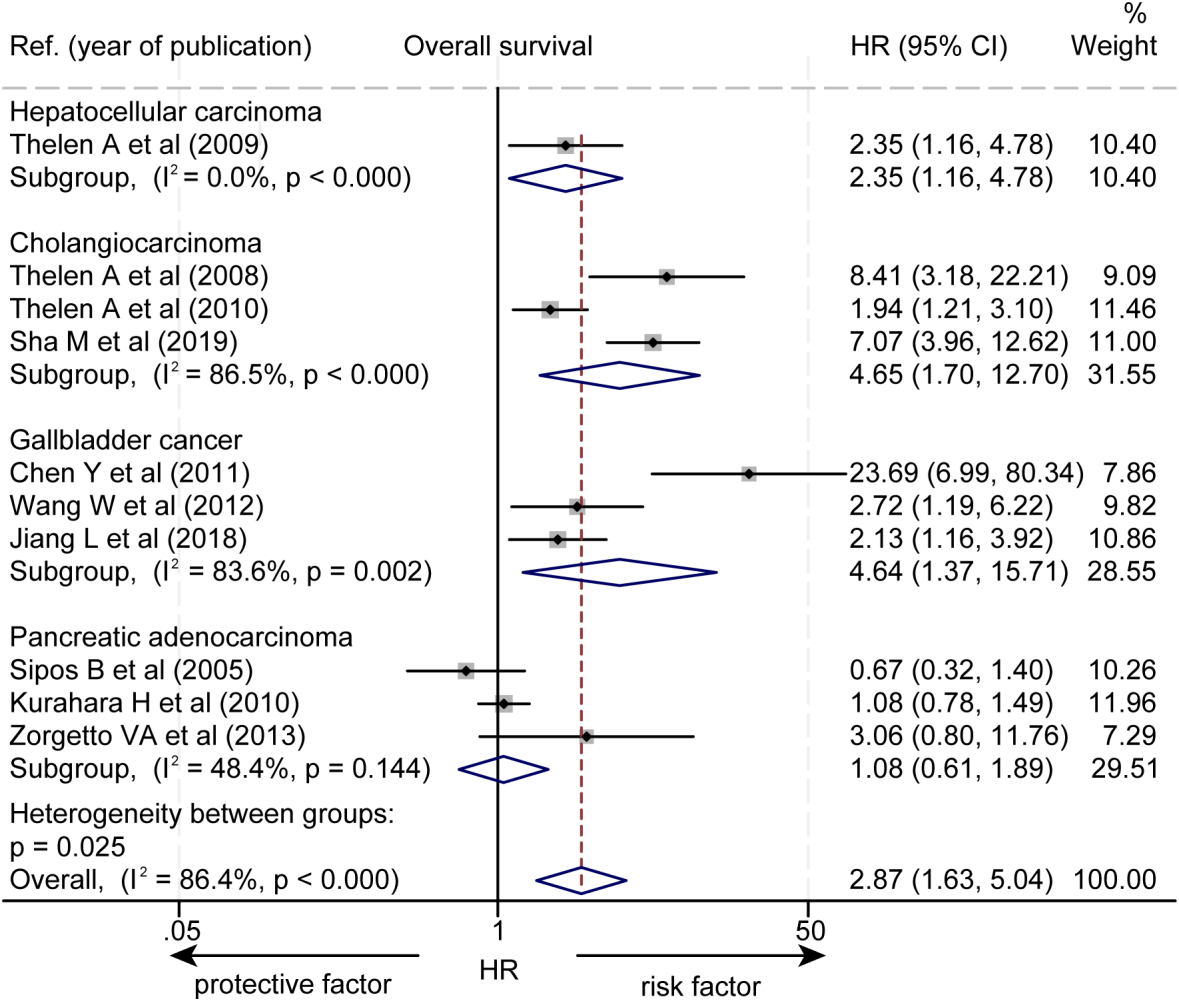
Subgroup forest plot of the impact of levels of LVD on overall survival of patients with hepatocellular carcinoma, cholangiocarcinoma, gallbladder cancer, or pancreatic cancer. CI, confidence interval; HR, hazard ratio; LVD, lymphatic vessel density.

Subgroup analysis based on pathological types revealed that a higher level of LVD was significantly associated with worse OS in HCC (HR = 2.35, 95% CI 1.16 to 4.78, **Figure 3**), in cholangiocarcinoma (HR = 4.65, 95% CI 1.70 to 12.70), and in gallbladder cancer (HR = 4.64, 95% CI 1.37 to 15.71). However, the levels of LVD were not significantly associated with OS in pancreatic cancer (HR = 1.08, 95% CI 0.61 to 1.89).

### Recurrence-free survival

**Figure 4:**
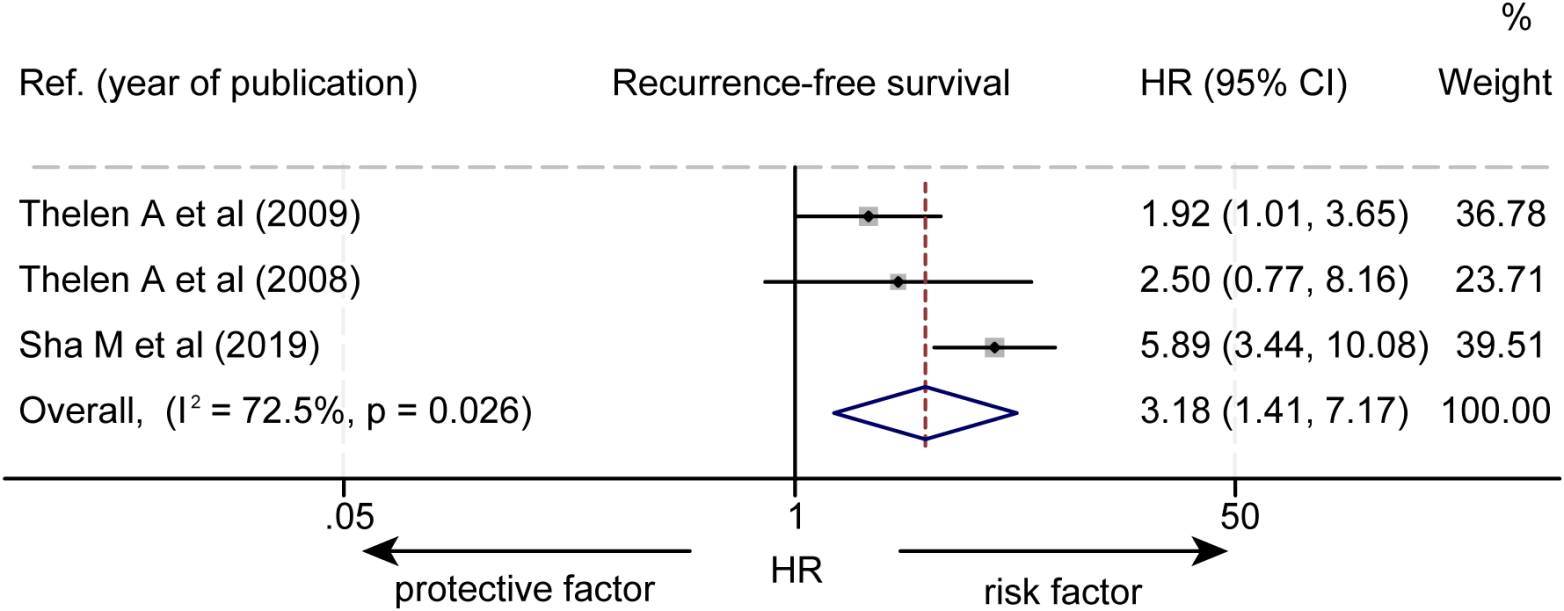
Forest plot of the impact of levels of LVD on recurrence-free survival of patients with hepato-biliary-pancreatic cancers after radical resection. CI, confidence interval; HR, hazard ratio; LVD, lymphatic vessel density.

**Figure 5:**
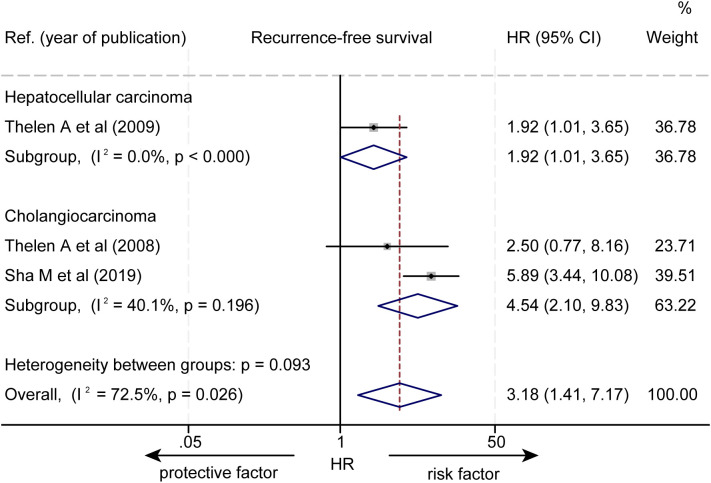
Subgroup forest plot of the impact of levels of LVD on recurrence-free survival of patients with hepatocellular carcinoma and cholangiocarcinoma. CI, confidence interval; HR, hazard ratio; LVD, lymphatic vessel density.

The effect of LVD on RFS was assessed in 3 studies (**Table 3**) (14–16). Two studies reported that a higher level of LVD in tumor was significantly associated with worse RFS (14, 15). One study reported that levels of LVD in tumor were not significantly associated with RFS (16). Data pooled from those 3 studies indicated that a higher level of LVD in tumor was significantly associated with worse RFS (HR = 3.18, 95% CI 1.41 to 7.17, **Figure 4**). Furthermore, a higher level of LVD was significantly associated with worse RFS in HCC (HR = 1.92, 95% CI 1.01 to 3.65, **Figure 5**) and in cholangiocarcinoma (HR = 4.54, 95% 2.10 to 9.83).

**Table 3:** The recurrence-free survival of patients with hepato-biliary-pancreatic cancers with higher and lower levels of lymphatic vessel density after radical resection.

Ref.	Publication year	Group	Recurrence-free survival		
			One-year	Three-year	Five-year
Thelen A et al. (14)	2009	High	38.00%	24.00%	18.00%
		Low	66.00%	46.00%	40.00%
Thelen A et al. (16)	2008	High	43.30%	8.30%	5.90%
		Low	95.20%	90.50%	76.40%
Sha M et al. (15)	2019	High	4.00%	2.00%	2.00%
		Low	64.30%	53.80%	50.40%

### Association between the levels of LVD and T stages

**Figure 6:**
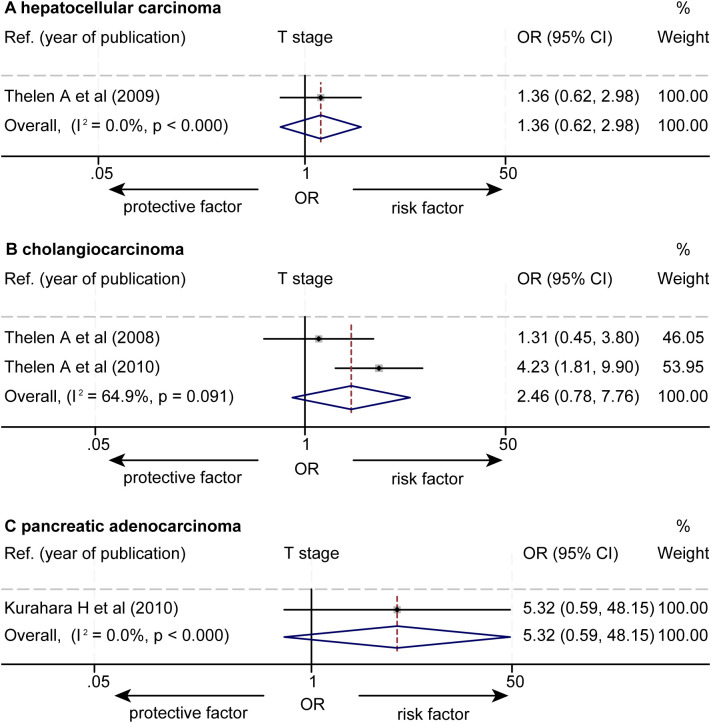
Forest plot of the impact of levels of LVD on T stage of patients with hepato-biliary-pancreatic cancers after radical resection. **(A)** hepatocellular carcinoma. **(B)** cholangiocarcinoma. **(C)** pancreatic adenocarcinoma. CI, confidence interval; LVD, lymphatic vessel density; OR, odds ratio.

The data about the association between the levels of LVD and T stages were extracted from 4 studies (**Supplementary Table S3**) (14, 16, 17, 21). One study reported that a higher level of LVD was significantly associated with higher T stages (17). Other three studies reported that the levels of LVD were not associated with T stages (14, 16, 21). The pooled data from the four studies unveiled that the levels of LVD were not associated with T stages in HCC (OR = 1.36, 95% CI 0.62 to 2.98, **Figure 6**) (14), in cholangiocarcinoma (OR = 2.46, 95% CI 0.78 to 7.76) (16, 17), and in pancreatic adenocarcinoma (OR = 5.32, 95% CI 0.59 to 48.15) (21).

### Association between the levels of LVD and lymph node metastasis

**Figure 7:**
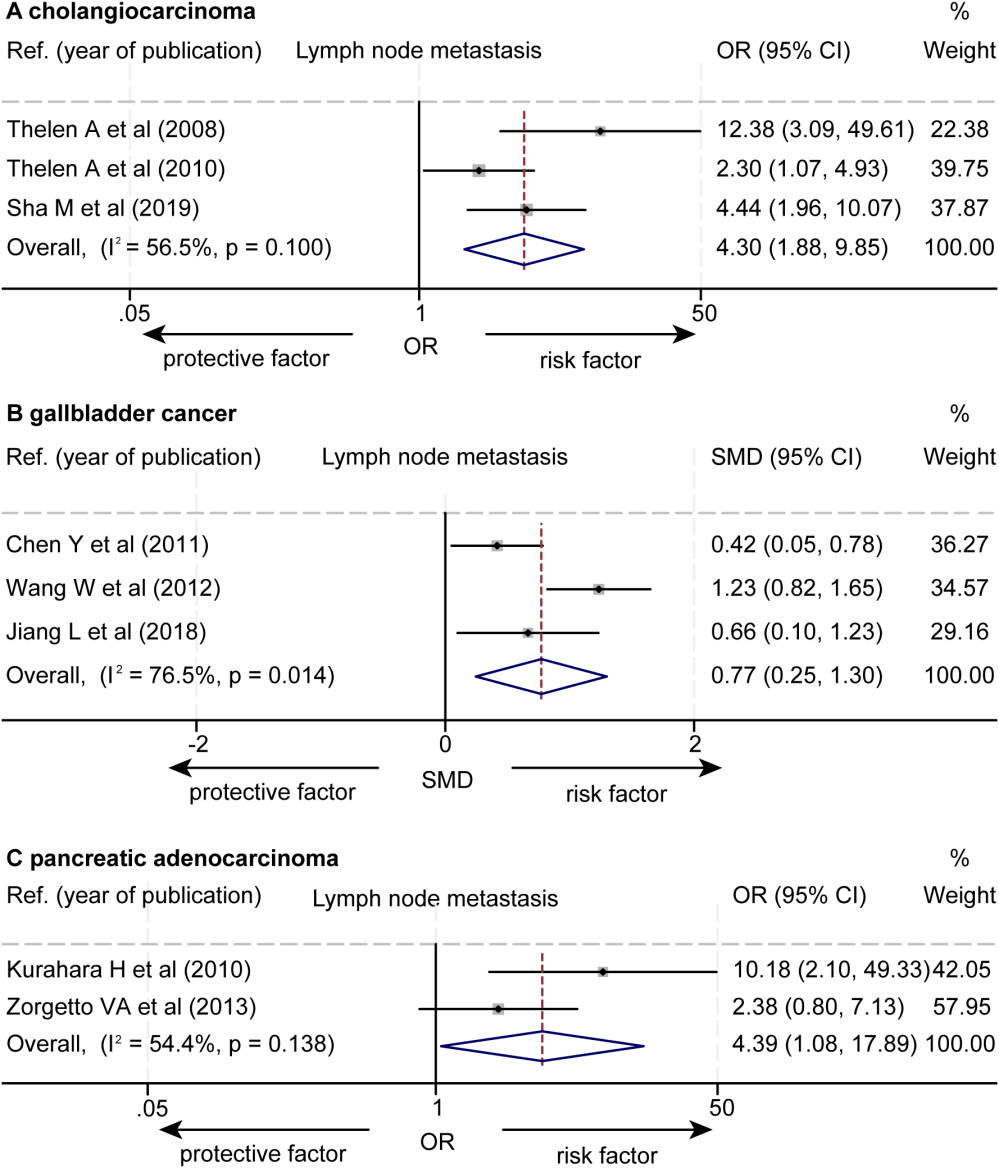
Forest plot of the impact of levels of LVD on lymph node metastasis of patients with hepato-biliary-pancreatic cancers after radical resection. **(A)** cholangiocarcinoma. **(B)** gallbladder cancer. **(C)** pancreatic adenocarcinoma. CI, confidence interval; LVD, lymphatic vessel density; OR, odds ratio. SMD, standardized mean difference.

The data about the association between the levels of LVD and lymph node metastasis were extracted from eight studies (**Supplementary Tables S4**, **S5**) (15–21, 23). Seven studies reported that a higher level of LVD was significantly associated with lymph node metastasis (15–21), while one study reported that the levels of LVD were not significantly associated with lymph node metastasis (23). The pooled data from the eight studies indicated that a higher level of LVD was significantly associated with lymph node metastasis in cholangiocarcinoma (OR = 4.30, 95% CI 1.88 to 9.85, **Figure 7**) (15–17), in gallbladder cancers (SMD = 0.77, 95% CI 0.25 to 1.30) (18–20), and in pancreatic adenocarcinoma (OR = 4.39, 95% CI 1.08 to 17.89) (21, 23).

### Association between the levels of LVD and differentiation grades

**Figure 8:**
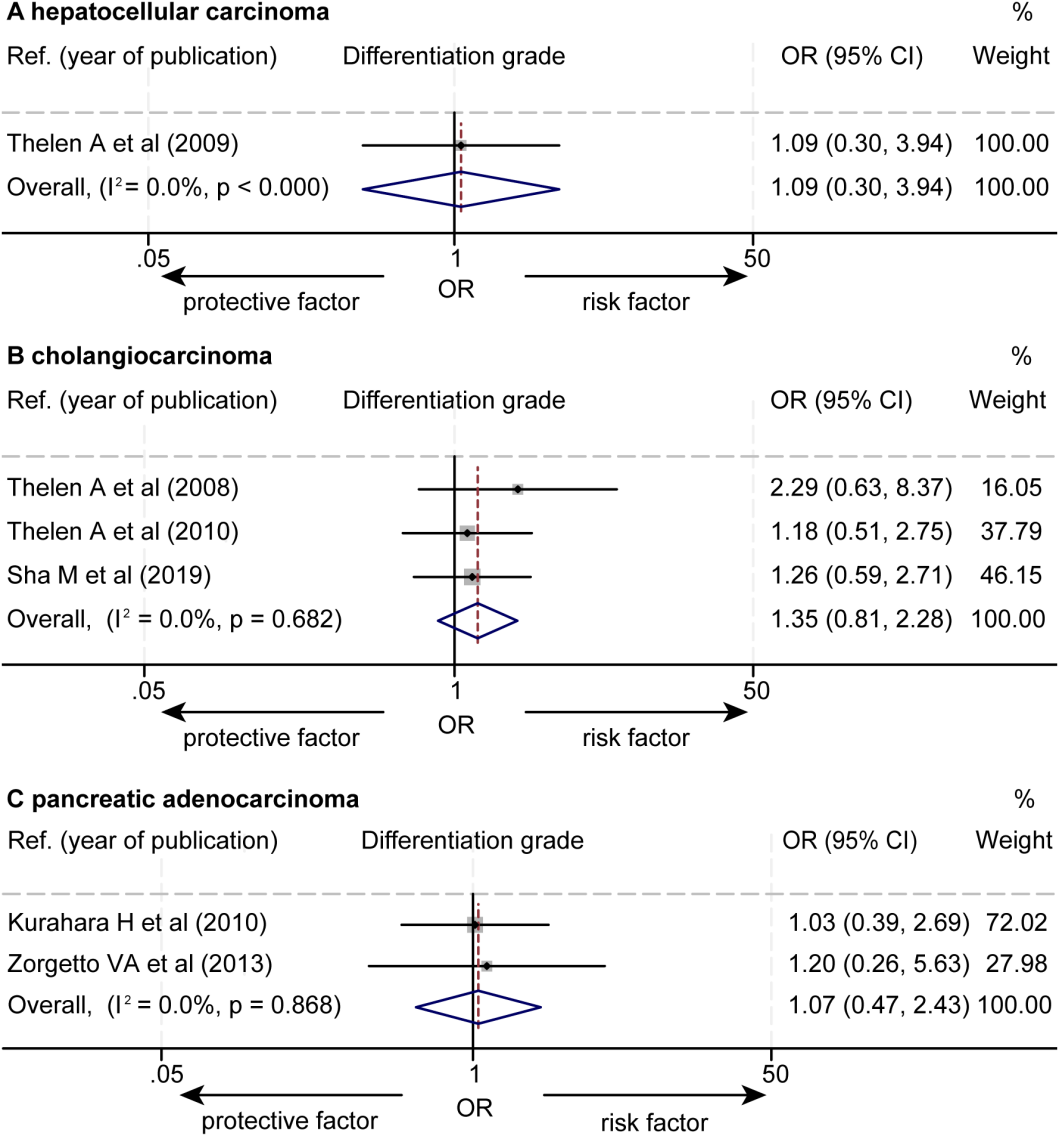
Forest plot of the impact of levels of LVD on cell differentiation grades of patients with hepato-biliary-pancreatic cancers after radical resection. **(A)** hepatocellular carcinoma. **(B)** cholangiocarcinoma. **(C)** pancreatic adenocarcinoma. CI, confidence interval; LVD, lymphatic vessel density; OR, odds ratio.

The data about the association between the levels of LVD and differentiation grades were extracted from six studies (**Supplementary Table S6**) (14–17, 21, 23). These six studies consistently reported that the levels of LVD were not significantly associated with tumor cell differentiation grades. The pooled data from these six studies revealed that the levels of LVD were not significantly associated with tumor cell differentiation grades in HCC (OR = 1.09, 95% CI 0.30 to 3.94, **Figure 8**) (14), cholangiocarcinoma (OR = 1.35, 95% CI 0.81 to 2.28) (15–17), and pancreatic adenocarcinoma (OR = 1.07, 95% CI 0.47 to 2.43) (21, 23).

### Publication bias

**Figure 9:**
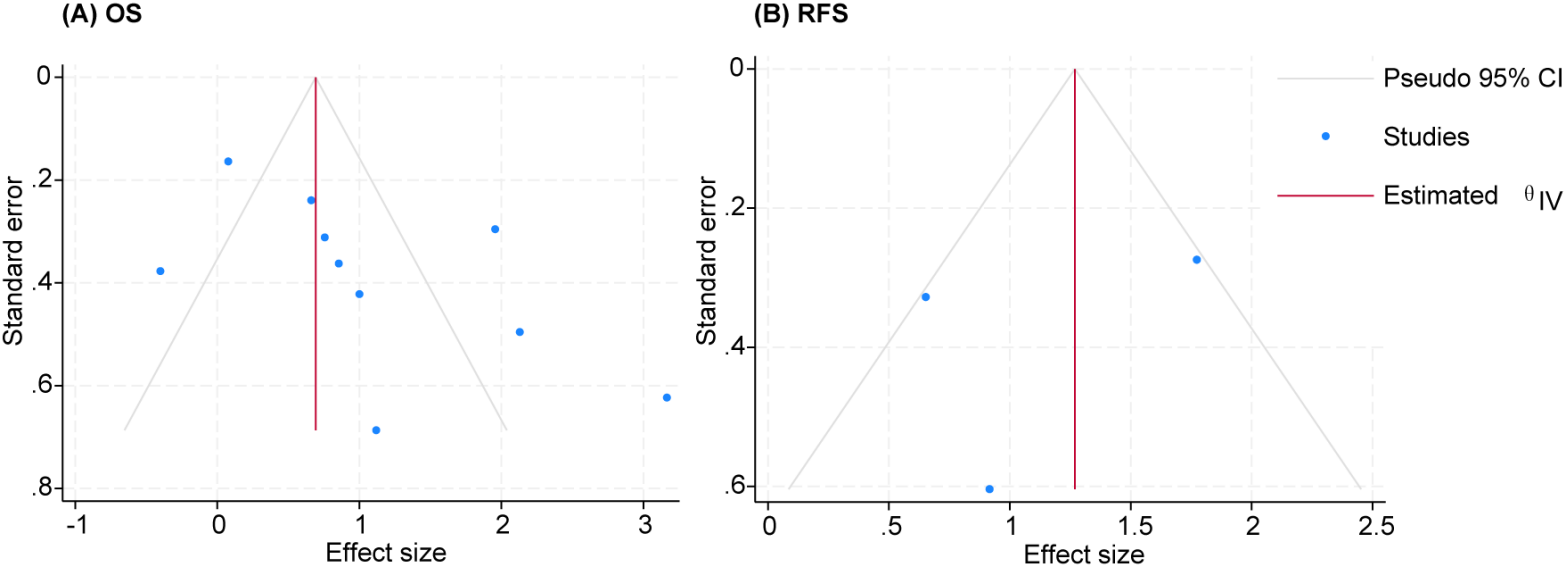
Funnel plot. **(A)** OS. **(B)** RFS. OS, overall survival; RFS, recurrence-free survival.

There was no significant publication bias in funnel plots for OS (Egger’s P = 0.0502 and Begg’s P = 0.1074, **Figure 9A**) or RFS (Egger’s P = 0.6316 and Begg’s P = 1.0000, **Figure 9B**).

## Discussion

The HBP system produces more than 50% of lymph passing through the thoracic duct (6, 28). The lymphatic vasculature in the HBP system is important for the maintenance of tissue fluid homeostasis, lipid metabolism, and immune surveillance (6, 7). Tumor-associated lymphatic vessels connect tumor with the enormous lymphatic vasculatures in the HBP system. In recent years (2005-2024), although some advances have been made in the study of the role of tumor-associated lymphangiogenesis in the prognosis of patients with HBP cancers after radical resection (14–23), the results remain inconclusive and controversial because of relatively small sample size and no prospective study. Currently, there is no systematic review and meta-analysis for this research gap. Therefore, we conducted this systematic review and meta-analysis to give some pieces of high-grade evidence for this topic.

**Figure 10:**
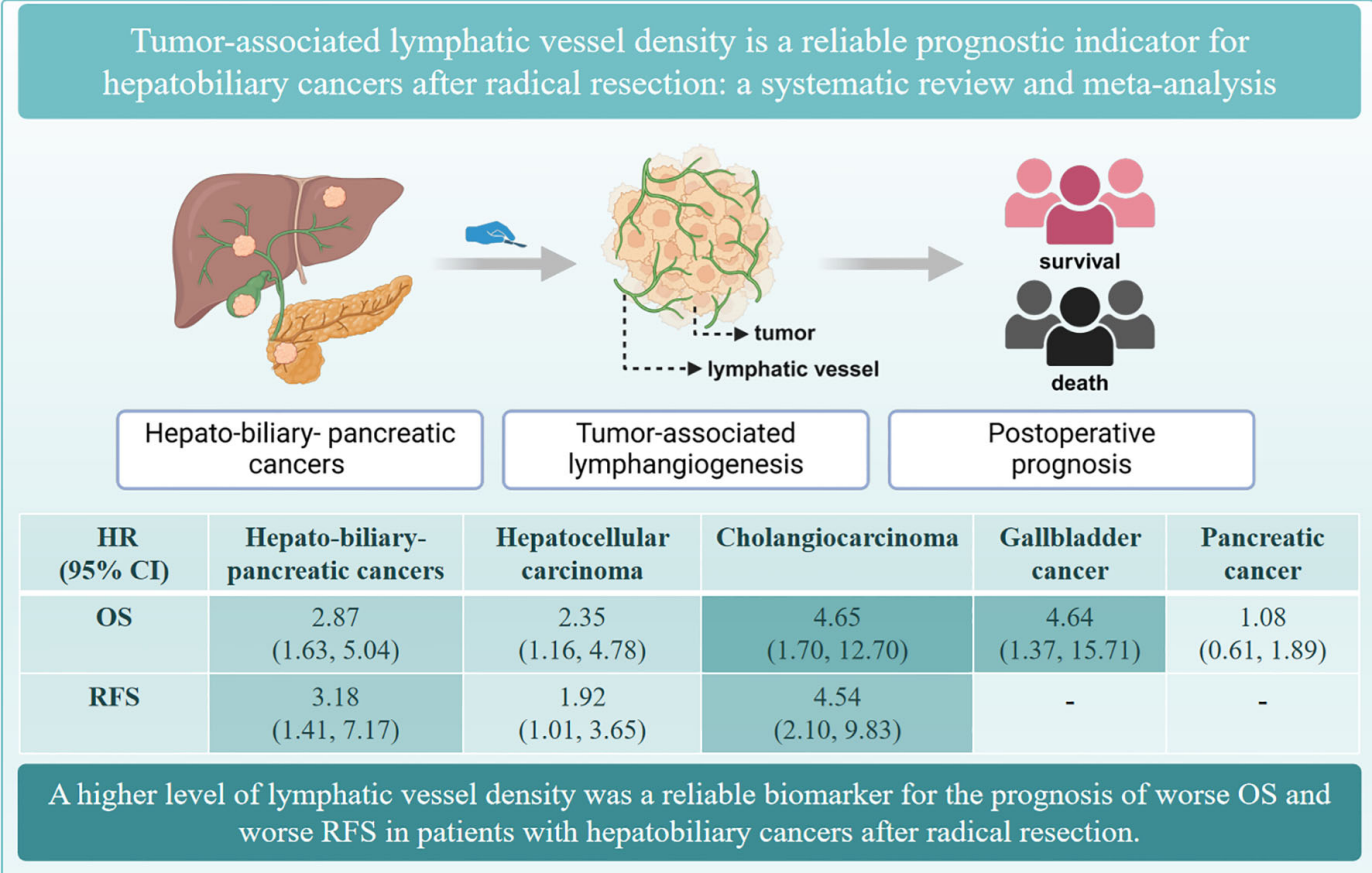
The summary of the role of tumor-associated lymphatic vessel density in the prognosis of hepato-biliary-pancreatic cancers.

Our findings revealed that patients with HBP cancers with a higher level of LVD in tumor had worse OS and worse RFS after radical resection than those with a lower level of LVD (**Figure 10**). Subgroup analysis indicated that a higher level of LVD was associated with worse OS and worse RFS in HCC, cholangiocarcinoma, and gallbladder cancer, but not associated with OS in pancreatic cancer. Hence, tumor-associated lymphangiogenesis had an obviously unfavorable impact on the prognosis of patients with hepatobiliary cancer, but not with pancreatic cancer after radical resection.

Consistently, tumor-associated lymphatic vessels can serve as metastatic conduits of hepatobiliary cancer cells to lymph nodes (29), and high levels of LVD can increase the probability of tumor cells invading lymph vessels and metastasizing to lymph nodes (8). This event dramatically influences the patient’s survival (30). Indeed, we found that patients with a higher level of LVD had more lymph node metastasis than those with a lower level of LVD. Therefore, tumor-associated lymphangiogenesis may be crucial for lymphatic vessel invasion and lymph node metastasis (8). Further prospective studies are needed to verify the opinion through determining the role of LVD in the recurrence or survival of patients with hepatobiliary cancer after radical resection when lymphatic vessel invasion or lymph node metastasis is not found.

On the other hand, tumor-associated lymphatic vessels have a variety of immunoregulatory functions to promote the growth and metastasis of hepatobiliary cancer by expressing a wide range of chemokines and receptors (6, 28). For example, IL-17A secreted by lymphatic endothelial cells promotes the self-renewal and immune escape of hepatoma stem cells in liver cancer (31). CXCL5 secreted by inflammatory lymphatic endothelial cells promotes migration, invasion, and metabolic reprogramming in cholangiocarcinoma, which further enhance the metastasis of cholangiocarcinoma (32).

Unexpectedly, subgroup analysis unveiled that patients with pancreatic adenocarcinoma with a higher level of LVD in tumor had similar OS to those with a lower level of LVD, although a higher level of LVD in tumor was significantly associated with lymph node metastasis. Two studies have explained these anomalies: one study has shown that although both a higher level of LVD and lymph node metastasis are significantly associated with worse OS by the Log-rank test, only lymph node metastasis, but not LVD, is the independent prognostic factor for worse OS by Cox’s proportional hazard regression analysis, highlighting that lymph node metastasis is a confounding variable between LVD and the prognosis of pancreatic adenocarcinoma (21). Another study has found that the rapid growth of tumors in advanced or terminal stages can lead to the collapse of lymphatic vessels, which may result in lower levels of LVD detection in pancreatic adenocarcinomas at these stages (23). Further prospective studies are needed to verify the role of LVD in the prognosis of patients with pancreatic cancer after radical resection.

The study had some limitations. Firstly, all data came from retrospective cohort studies. Secondly, the categories for “high” and “low” levels of LVD were inconsistent for all selected studies, which may increase the heterogeneity in the meta-analysis. Thirdly, the selected studies were from four countries, which may not be completely representative of the world population and may reduce the generalizability of our findings. Lastly, there was only one study with 60 HCC patients in this meta-analysis. Moreover, the sample sizes of cholangiocarcinoma, gallbladder cancer, and pancreatic adenocarcinoma groups were relatively small. It is needed to conduct more prospective, multi-center studies with larger sample sizes to verify our findings in the future.

## Conclusions

In conclusion, patients with hepatobiliary cancers with a higher level of LVD had worse OS and RFS after radical resection than those with a lower level of LVD. LVD is a reliable biomarker for the prognosis of patients with hepatobiliary cancers after radical resection.

## Data Availability

The original contributions presented in the study are included in the article/**Supplementary Material**. Further inquiries can be directed to the corresponding authors.
