# Supplementary material for: Tumor-associated lymphatic vessel density is a postoperative prognostic biomarker of hepatobiliary cancers: a systematic review and meta-analysis
Source: Front Immunol. 2025 Jan 7;15:1519999. doi: 10.3389/fimmu.2024.1519999 (PMC11747820; doi:10.3389/fimmu.2024.1519999)
Supplement: Supplementary file 1 [file DataSheet1.doc]

**Tumor-associated lymphatic vessel density is a postoperative prognostic biomarker of hepatobiliary cancers: a systematic review and meta-analysis**

Jin Li, Yu-Bo Liang, Qing-Bo Wang, Yu-Kai Li, Xing-Ming Chen, Wan-Ling Luo, Yawhan Lakang, Zi-Sheng Yang, Yan Wang, Zhi-Wei Li, Yang Ke

**Supplementary material**

**Table S1** Search terms for hepato-biliary-pancreatic cancers.

**Table S2** Quality assessment for included studies.

**Table S3** Association between lymphatic vessel density and T stages of hepato-biliary-pancreatic cancers.

**Table S4** Association between lymphatic vessel density and lymph node metastasis of cholangiocarcinoma and pancreatic adenocarcinoma.

**Table S5** Association between lymphatic vessel density and lymph node metastasis of gallbladder cancer.

**Table S6** Association between lymphatic vessel density and differentiation grades of hepato-biliary-pancreatic cancers.

**Table S1 Search terms for hepato-biliary-pancreatic cancers.**

| Cancer types | Search terms |
| --- | --- |
| Hepatocellular carcinoma | (lymphangiogenesis OR lymphangiogeneses OR (lymph vessel density) OR (lymphatic vessel density) OR (lymph microvessel density) OR (lymphatic microvessel density) OR (lymph vascular density) OR (lymphatic vascular density) OR podoplanin OR D2-40) AND ((hepatocellular carcinoma) OR (hepatocellular carcinomas) OR hepatoma OR hepatomas OR (liver cancer) OR (liver cancers) OR (liver carcinoma) OR (liver carcinomas) OR (liver cell cancer) OR (liver cell cancers) OR (liver cell carcinoma) OR (liver cell carcinomas)) |
| Cholangiocarcinoma | (lymphangiogenesis OR lymphangiogeneses OR (lymph vessel density) OR (lymphatic vessel density) OR (lymph microvessel density) OR (lymphatic microvessel density) OR (lymph vascular density) OR (lymphatic vascular density) OR podoplanin OR D2-40) AND (cholangiocarcinoma OR cholangiocarcinomas OR (cholangiocellular carcinoma) OR (cholangiocellular carcinomas) OR (Klatskin tumor) OR (Klatskin tumors) OR (Klatskin’s tumor) OR (Klatskin’s tumors) OR (biliary neoplasm) OR (biliary neoplasms) OR (biliary cancer) OR (biliary cancers) OR (biliary carcinoma) OR (biliary carcinomas) OR (biliary adenocarcinoma) OR (biliary adenocarcinomas) OR (biliary duct neoplasm) OR (biliary duct neoplasms) OR (biliary duct cancer) OR (biliary duct cancers) OR (biliary duct carcinoma) OR (biliary duct carcinomas) OR (biliary duct adenocarcinoma) OR (biliary duct adenocarcinomas) OR (biliary tract neoplasm) OR (biliary tract neoplasms) OR (biliary tract cancer) OR (biliary tract cancers) OR (biliary tract carcinoma) OR (biliary tract carcinomas) OR (biliary tract adenocarcinoma) OR (biliary tract adenocarcinomas)) |
| Gallbladder cancer | (lymphangiogenesis OR lymphangiogeneses OR (lymph vessel density) OR (lymphatic vessel density) OR (lymph microvessel density) OR (lymphatic microvessel density) OR (lymph vascular density) OR (lymphatic vascular density) OR podoplanin OR D2-40) AND ((gallbladder neoplasm) OR (gallbladder neoplasms) OR (gall bladder neoplasm) OR (gall bladder neoplasms) OR (gallbladder cancer) OR (gallbladder cancers) OR (gall bladder cancer) OR (gall bladder cancers) OR (gallbladder carcinoma) OR (gallbladder carcinomas) OR (gall bladder carcinoma) OR (gall bladder carcinomas) OR (gallbladder adenocarcinoma) OR (gallbladder adenocarcinomas) OR (gall bladder adenocarcinoma) OR (gall bladder adenocarcinomas) OR (gallbladder squamous carcinoma) OR (gallbladder squamous carcinomas) OR (gall bladder squamous carcinoma) OR (gall bladder squamous carcinomas) OR (gallbladder adenosquamous carcinoma) OR (gallbladder adenosquamous carcinomas) OR (gall bladder adenosquamous carcinoma) OR (gall bladder adenosquamous carcinomas)) |
| Pancreatic cancer | (lymphangiogenesis OR lymphangiogeneses OR (lymph vessel density) OR (lymphatic vessel density) OR (lymph microvessel density) OR (lymphatic microvessel density) OR (lymph vascular density) OR (lymphatic vascular density) OR podoplanin OR D2-40) AND ((pancreatic neoplasm) OR (pancreatic neoplasms) OR (pancreas neoplasm) OR (pancreas neoplasms) OR (pancreatic cancer) OR (pancreatic cancers) OR (pancreas cancer) OR (pancreas cancers) OR (pancreatic carcinoma) OR (pancreatic carcinomas) OR (pancreas carcinoma) OR (pancreas carcinomas) OR (pancreatic duct carcinoma) OR (pancreatic duct carcinomas) OR (pancreatic ductal carcinoma) OR (pancreatic ductal carcinomas) OR (pancreatic adenocarcinoma) OR (pancreatic adenocarcinomas) OR (pancreatic duct adenocarcinoma) OR (pancreatic duct adenocarcinomas) OR (pancreatic ductal adenocarcinoma) OR (pancreatic ductal adenocarcinomas) OR (cancer of pancreas) OR (cancers of pancreas)) |

**Table S2** Quality assessment for included studies

| Study | Selection (4 points) | | | | Comparability (2 points) | Exposure (3 points) | | | Overall (9 points) |
| --- | --- | --- | --- | --- | --- | --- | --- | --- | --- |
|  | Representative of the Exposed Cohort | Selection of Non-Exposed | Ascertainment of Exposure | Outcome Not Present at Start |  | Assessment of Outcome | Adequate Follow-Up Length | Adequacy of Follow-Up |  |
| Thelen A et al 2009 (14) | * | * | * | * | * | * | * | * | 8/9 |
| Thelen A et al 2008 (16) | * | * | * | * | * | * | * | * | 8/9 |
| Thelen A et al 2010 (17) | * | * | * | * | * | * | * | * | 8/9 |
| Sha M et al 2019 (15) | * | * | * | * | * | * | * | * | 8/9 |
| Chen Y et al 2011 (19) | * | * | * | * | * |  |  | * | 6/9 |
| Wang W et al 2012 (18) | * | * | * | * | * | * |  | * | 7/9 |
| Jiang L et al 2018 (20) | * | * | * | * | * | * |  | * | 7/9 |
| Sipos B et al 2005 (22) | * | * | * | * | * | * |  | * | 7/9 |
| Kurahara H et al 2010 (21) | * | * | * | * | * | * |  | * | 7/9 |
| Zorgetto VA et al 2013 (23) | * | * | * | * | * |  |  | * | 6/9 |

**Table S3** Association between lymphatic vessel density and T stages of hepato-biliary-pancreatic cancers.

| Ref. | Publication year | Pathological diagnosis | Exposure group | T3/T4 stages (n) | T1/T2 stages (n) |
| --- | --- | --- | --- | --- | --- |
| Thelen A et al (14) | 2009 | Hepatocellular carcinoma | High | 31 | 15 |
|  |  |  | Low | 41 | 27 |
| Thelen A et al (16) | 2008 | Hilar cholangiocarcinoma | High | 23 | 11 |
|  |  |  | Low | 16 | 10 |
| Thelen A et al (17) | 2010 | Intrahepatic cholangiocarcinoma | High | 41 | 10 |
|  |  |  | Low | 31 | 32 |
| Kurahara H et al (21) | 2010 | Pancreatic adenocarcinoma | High | 33 | 1 |
|  |  |  | Low | 31 | 5 |

**Table S4** Association between lymphatic vessel density and lymph node metastasis of cholangiocarcinoma and pancreatic adenocarcinoma.

| Ref. | Publication year | Pathological diagnosis | Exposure group | Positive lymph node metastasis (n) | Negative lymph node metastasis (n) |
| --- | --- | --- | --- | --- | --- |
| Thelen A et al (16) | 2008 | Hilar cholangiocarcinoma | High | 21 | 13 |
|  |  |  | Low | 3 | 23 |
| Thelen A et al (17) | 2010 | Intrahepatic cholangiocarcinoma | High | 27 | 19 |
|  |  |  | Low | 26 | 42 |
| Sha M et al (15) | 2019 | Intrahepatic cholangiocarcinoma | High | 32 | 18 |
|  |  |  | Low | 16 | 40 |
| Kurahara H et al (21) | 2010 | Pancreatic adenocarcinoma | High | 32 | 2 |
|  |  |  | Low | 22 | 14 |
| Zorgetto VA et al (23) | 2013 | Pancreatic adenocarcinoma | High | 11 | 10 |
|  |  |  | Low | 12 | 26 |

**Table S5 Association between lymphatic vessel density and lymph node metastasis of gallbladder cancer.**

| Ref. | Publication year | Pathological diagnosis | Group | No. of patients (n) | Mean of LVD (/HPF) | Standard deviation of LVD |
| --- | --- | --- | --- | --- | --- | --- |
| Chen Y et al (19) | 2011 | Gallbladder cancer | Positive lymph node metastasis | 54 | 14.07 | 2.32 |
|  |  |  | Negative lymph node metastasis | 64 | 13.07 | 2.44 |
| Wang W et al (18) | 2012 | Gallbladder cancer | Positive lymph node metastasis | 59 | 14.40 | 5.40 |
|  |  |  | Negative lymph node metastasis | 49 | 7.80 | 5.20 |
| Jiang L et al (20) | 2018 | Gallbladder cancer | Positive lymph node metastasis | 27 | 8.00 | 3.80 |
|  |  |  | Negative lymph node metastasis | 24 | 5.70 | 2.90 |

HPF, high-power field in microscopy

**Table S6** Association between lymphatic vessel density and differentiation grades of hepato-biliary-pancreatic cancers.

| Ref. | Publication year | Pathological diagnosis | Exposure group | Poor differentiation (n) | Well or moderate differentiation (n) |
| --- | --- | --- | --- | --- | --- |
| Thelen A et al (14) | 2009 | Hepatocellular carcinoma | High | 5 | 19 |
|  |  |  | Low | 7 | 29 |
| Thelen A et al (16) | 2008 | Hilar cholangiocarcinoma | High | 10 | 24 |
|  |  |  | Low | 4 | 22 |
| Thelen A et al (17) | 2010 | Intrahepatic cholangiocarcinoma | High | 13 | 33 |
|  |  |  | Low | 17 | 51 |
| Sha M et al (15) | 2019 | Intrahepatic cholangiocarcinoma | High | 27 | 23 |
|  |  |  | Low | 27 | 29 |
| Kurahara H et al (21) | 2010 | Pancreatic adenocarcinoma | High | 21 | 13 |
|  |  |  | Low | 22 | 14 |
| Zorgetto VA et al (23) | 2013 | Pancreatic adenocarcinoma | High | 3 | 17 |
|  |  |  | Low | 5 | 34 |
